# Conception and fertility: A survey examining the practices, attitudes, and knowledge of reproductive-age Greek women

**DOI:** 10.18332/ejm/200843

**Published:** 2025-03-26

**Authors:** Evangelia Saranti, Vicentia C. Harizopoulou, Viktoria Christoforaki, Eleni Bili, George Pados, Dimitrios G. Goulis, Dimitrios Vavilis

**Affiliations:** 1 First Department of Obstetrics and Gynecology, Papageorgiou General Hospital, Medical School, Aristotle University of Thessaloniki, Thessaloniki, Greece; 2 Department of Midwifery, University of West Attica, Athens, Greece; 3 Medical School, University of Cyprus, Nicosia, Cyprus

**Keywords:** attitudes, fertility, awareness, reproductive behavior, health knowledge, parenthood intention

## Abstract

**INTRODUCTION:**

There is a growing global concern about contemporary behavior regarding fertility and fertility awareness. This study investigated the knowledge, attitudes and practices regarding conception and fertility among Greek women of reproductive age.

**METHODS:**

An online survey of Greek women (aged 18–40 years) was conducted. The questionnaire included 57 questions regarding childbearing intention, knowledge about fertility, aging, ovulation, sources of information about fertility, attitudes and knowledge regarding assisted reproductive techniques (ART).

**RESULTS:**

The survey included 817 women (aged 18–25 years: 33.2%, 26–30 years: 24.8%, 31–35 years: 18%, and 36–40 years: 24%). The participants were married (24.7%) or in a stable relationship (38.3%); 90.1% intended to have children. Regarding knowledge related to fertility, 59.5% of the responses were correct, 19.3% incorrect and 21.2% were ‘unaware’. The average score was 19.6 ± 4.8 (range: 0–32). High fertility awareness was recorded for the topic ‘how woman’s aging affects conception and pregnancy’ (73.7%). Moderate and low fertility awareness was recorded for the topics ‘ovulation, folic acid and conception’ (51.5%) and ‘how man’s aging affects conception and pregnancy’ (38.7%), respectively. Regarding attitudes and practices, 59.1% reported intention to undergo ART if they failed to achieve pregnancy, and 53.4% had never discussed fertility problems with a healthcare professional.

**CONCLUSIONS:**

The knowledge of Greek women aged 18–40 years regarding reproductive health and conception is moderate to high. Significant knowledge gaps and misconceptions were identified in certain fertility topics. Central planning on fertility education in Greece is needed to improve fertility awareness.

## INTRODUCTION

In developed countries worldwide, women postpone childbearing to after 30 years of age^1^. Within the European Union (27 countries, 2020), the mean age of having the first child was 29.5 years, and the mean childbirth age was 31 years^2^. Respective means for Greece were 30.7 and 31.7 years^2^. Women who postpone childbearing face the risk of infertility due to age-related fertility decline and increased risk for maternal and neonatal complications (spontaneous miscarriage, preterm labor, gestational diabetes mellitus, pre-eclampsia, stillbirth, chromosomal abnormalities, and cesarean delivery)^3^. Moreover, delayed childbearing contributes to a growing need for infertility treatment and assisted reproductive techniques (ART)^4^.

Fertility and fertility awareness affect multiple demographic parameters. Globally, there is growing evidence on the determinants and the consequences of reproductive behavior. Several studies investigated the knowledge, attitudes, misconceptions, and practices adopted by women and of the underlying lifestyle (body mass index, smoking, alcohol consumption) and sociocultural factors (marital status, religion, education, employment, and financial factors) that can influence women’s reproductive choices^5-8^.

A way to optimize reproductive and pregnancy outcomes is to identify gaps in knowledge, attitudes, and practices among women of reproductive age. Then, targeted educational interventions can be developed by assessing fertility awareness before planning a pregnancy and conception. A recent systematic review suggests that fertility awareness among reproductive-age people is low to moderate globally^8^. Two relevant Greek studies have been conducted: a cross-sectional study regarding medical students’ knowledge and attitudes towards infertility and ART^9^, and an online survey of men and women aged 18–26 years regarding information on the intention to parenthood and knowledge on fertility issues^10^. The present study investigated the knowledge, attitudes, and practices regarding conception and fertility among Greek women of reproductive age.

## METHODS

### Participants and study design

A cross-sectional study was performed in 2021, with the use of an online survey forwarded to a convenience sample of Greek women (aged 18–40 years). The study complied with all the relevant national regulations and institutional policies and followed the tenets of the Helsinki Declaration. The study protocol was approved by the corresponding Committees of the Medical School, Aristotle University of Thessaloniki, Greece (board meeting number 11/27 November 2018). All women were informed in detail regarding the objective of the survey on the first page of the online questionnaire. A ‘click-if-you-agree’ button obtained informed consent. The questionnaire was completed by 817 Greek women aged 18–40 years. No personal identification information was collected, while incomplete questionnaires were not analyzed.

### Questionnaire development

The questionnaire included 57 questions regarding childbearing intention, knowledge about fertility, aging, ovulation, sources of information about fertility, attitudes and knowledge regarding ART. Our team, comprised academic medical doctors (endocrinologists and gynecologists) and academic midwives, who developed an online questionnaire by reviewing the factors influencing fertility and related information published in the literature^5,11-14^. A pilot survey was conducted on ten women who met the study entry criteria to ensure the questionnaire was understandable.

The questionnaire included 57 questions regarding: 1) childbearing intention (2 questions); 2) knowledge about fertility, aging, ovulation, folic acid administration, and conception (33 questions); 3) sources of information about fertility (11 questions); and 4) attitudes and knowledge regarding ART (4 questions). Some of them (fertility, aging, ovulation, folic acid, and conception) were organized into ‘fertility awareness topics’; the awareness of each topic was scored as the percentage of correct responses (low <40%, moderate 40–59%, high 60%)^8^.

### Statistical analysis

The sample size was calculated to be 800 participants, using 95% confidence intervals^15^. Descriptive statistics and further analyses were carried out by IBM SPSS ver. 22.0 (IBM Corp., Armonk, NY, USA). One-way ANOVA was used to determine if there was any difference between the knowledge scores of independent subgroups of the participants. Cramer’s V measure tested the correlation between two nominal variables. A p≤0.05 was considered significant.

## RESULTS

### Demographic and socioeconomic characteristics

The demographic and socioeconomic characteristics of the 817 participants are summarized in Table 1. Of the women, 33.2% were aged 18–25 years, 24.8% were 26–30 years, 18% were 31–35 years, and 24% were 36–40 years. The participants were married (24.7%) or in a stable relationship (38.3%). Of them, 53.1% were university graduates, and 28.4% had a Master’s/Doctorate degree. More than 70% were employed in the public or private sector or self-employed. In terms of personal monthly income (€): 16.3% declared 0; 18.2%, 1–500; 38.6%, 501–1000; 20.3%, 1001–2000; and 6.6%, >2000. Of the participants, 98% had Greek nationality, and 64.1% resided in Athens or Thessaloniki.

**Table 1:** Demographic and socioeconomic characteristics

*Characteristics*	*Categories*	*n (%)*
**Age** (years)	18–25	271 (33.2)
	26–30	203 (24.8)
	31–35	147 (18.0)
	36–40	196 (24.0)
**Marital status**	Married	202 (24.7)
	In a stable relationship	313 (38.3)
	In a non-stable relationship	70 (8.6)
	Single	232 (28.4)
**Education level**	High school	69 (8.4)
	Technical education	80 (9.8)
	College/university	434 (53.1)
	MSc/PhD	234 (28.6)
**Occupation**	Employee, public sector	174 (21.3)
	Employee, private sector	314 (38.4)
	Self-employed	92 (11.3)
	Student	187 (22.9)
	Unemployed	50 (6.1)
**Personal monthly income** (€)		
	0	133 (16.3)
	1–500	149 (18.2)
	501–1000	315 (38.6)
	1001–2000	166 (20.3)
	>2000	54 (6.6)
**Nationality**	Greek	801 (98.0)
	Other	16 (2.0)
**Residence**	Athens/Thessaloniki	524 (64.1)
	Urban area	84 (10.3)
	Semi-urban area	84 (10.3)
	Rural area	65 (8.0)
	Abroad	60 (7.3)

### Childbearing intention and factors that affect the childbearing intention

Most participants (77.1%) intend to have children in the future, 9.9% do not intend to have children, and 13% were trying to conceive at the time of the survey. Table 2 presents the degree of the effect of several factors on the participants’ childbearing intention. Childbearing intention was strongly correlated with age (Cramer’s V=0.31, p<0.001) and marital status (Cramer’s V=0.32, p<0.001). A moderate correlation was found between the intention to have children and education (Cramer’s V=0.10, p=0.007), occupation (Cramer’s V=0.17, p<0.001), and personal monthly income (Cramer’s V=0.15, p<0.001).

**Table 2:** Factors that affect the childbearing intention

*Factors*	*Not at all* *n (%)*	*A little* *n (%)*	*Very much* *n (%)*
Financial security	34 (4.2)	247 (30.2)	536 (65.6)
Marriage/stable relationship	27 (3.3)	97 (11.9)	693 (84.8)
Good health	10 (1.2)	79 (9.7)	728 (89.1)
Permanent job	23 (2.8)	197 (24.1)	597 (73.1)
Completed education	185 (22.6)	205 (25.1)	427 (52.3)

### Knowledge about fertility, aging, ovulation, folic acid and conception

**Figure 1:**
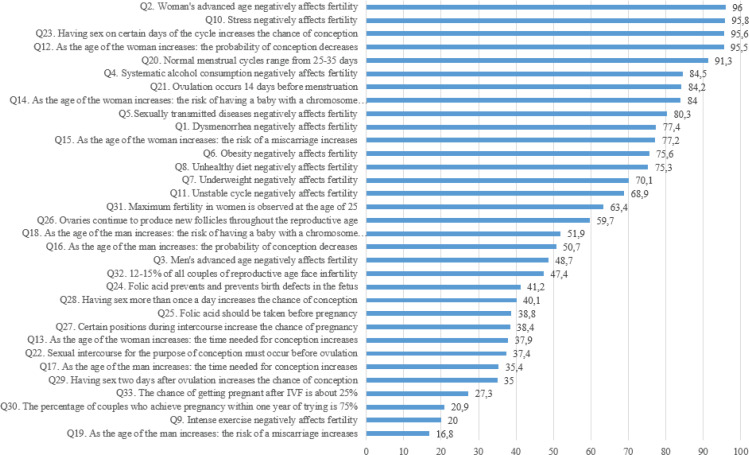
Percentage of correct answers to questions

Regarding knowledge related to fertility (33 questions), 59.5% of the responses (n=16035) were correct, 19.3% (n=5218) were incorrect, and 21.2% (n=5708) were ‘unaware’. The average score (number of correct responses) was 19.6 ± 4.8 (range: 0–32). There was a positive correlation between age group, personal monthly income, and education with score (p<0.001 for all, one-way ANOVA). Moreover, married women, employees, and those trying to conceive at the time of the survey obtained higher scores (p<0.001 for all, one-way ANOVA). Residence did not impact the score (p=0.139, one-way ANOVA). Supplementary file Table 1 presents the score per subgroup according to age, marital status, education level, occupation, personal monthly income, residence, and childbearing intention. Figure 1 presents the percentages of correct responses per question.

*Factors that negatively affect fertility*

Participants answered 11 questions about the factors that negatively affect fertility. The total percentage of correct responses was 66%. Most participants answered correctly about the negative effect of certain factors on fertility: woman’s advanced age (96%), systematic alcohol consumption (84.5%), sexually transmitted diseases (80.3%), obesity (75.6%), underweight (70.1%), unhealthy diet (75.3%), stress (95.8%), and irregular cycle (68.9%). Moreover, 77.4% of the participants knew that dysmenorrhea does not negatively affect fertility. On the contrary, only 48.7% and 20% of them responded correctly about the negative effect of a man’s advanced age and intense exercise on fertility, respectively.

*How the woman’s age affects conception and pregnancy*

Participants answered four questions about how a woman’s aging affects conception and pregnancy. The total percentage of correct responses on this topic was 73.65% (high). Most participants knew that as the woman’s age increases, the probability of conception decreases (95.5%), the risk of having a baby with a chromosome abnormality (84%) and the risk of a miscarriage (77.2%) increases. On the other hand, only 37.9% of the participants responded correctly that as the woman’s age increases, the time needed for conception increases.

*How the man’s age affects conception and pregnancy*

Participants answered four questions about how man’s aging affects conception and pregnancy. The total percentage of correct responses on this topic was 38.7% (low). Almost half of the participants (50.7%) knew that as the man’s age increases, the probability of conception decreases, and 51.9% knew that the risk of having a baby with a chromosome abnormality increases. On the other hand, only 35.4% and 16.8% of the participants responded correctly that as the age of the man increases, the time needed for conception increases, and the risk of a miscarriage increases, respectively.

*Ovulation, folic acid, and conception*

Participants answered 14 questions about ovulation, folic acid administration, and conception. The total percentage of correct responses on this topic was 51.5% (moderate). Most (91.3%) participants knew that regular menstrual cycles range from 25–35 days, and 84.2% knew that ovulation occurs 14 days before menstruation. Just 37.4% knew that sexual intercourse for conception must occur before ovulation, and 95.6% knew that having sex on certain days of the cycle increases the chance of conception. Four out of ten participants (41.2%) knew that folic acid prevents fetal congenital anomalies and should be taken before pregnancy (38.8%). Most knew that ovaries continue to produce new follicles throughout the reproductive age (59.7%) and that the maximum fertility in women is observed at the age of 25 years (63.4%). Only 38.4% of the participants responded correctly that certain positions during sexual intercourse do not increase the chance of pregnancy. In comparison, only 40.1% knew that having sexual intercourse more than once a day does not increase the likelihood of conception. Finally, 33.2% responded correctly that having sexual intercourse two days after ovulation does not increase the chance of conception, and 20.9% knew that the percentage of couples who achieve pregnancy within one year of trying is 75%.

### Beliefs and attitudes toward ART

Table 3 illustrates the responses to Greek women’s beliefs and attitudes toward ART. Almost six out of ten participants (59.1%) said that they would turn to ART if they failed to achieve pregnancy. Of the participants, 30.7% believe that in Greece, every woman has access to ART services. Almost half of the women stated they knew the cost of ART services. Finally, 59.2% knew some of the treatments offered in ART.

**Table 3:** Beliefs and attitudes toward ART

	*Yes* *n (%)*	*No* *n (%)*	*Unaware* *n (%)*
In case of failure to achieve pregnancy, I would turn to ART	483 (59.1)	146 (17.9)	188 (23.0)
In Greece, every woman has access to ART services	251 (30.7)	286 (35.0)	280 (34.3)
Do you know the cost of ART services?	406 (49.7)	222 (27.2)	189 (23.1)
Do you know some of the treatments offered in ART?	484 (59.2)	167 (20.4)	166 (20.3)

ART: assisted reproduction techniques.

### Sources to obtain information on conception and fertility

**Figure 2:**
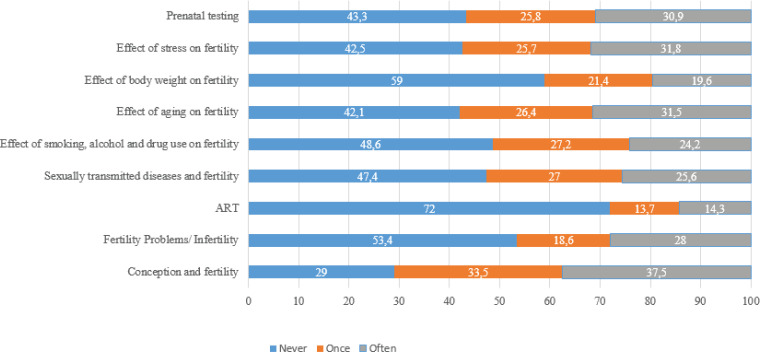
Answers to the question 'How often have you discussed the following topics with a healthcare professional?' (values are presented as %)

Table 4 presents the sources mentioned by the participants regarding obtaining information on conception and fertility. The majority never consulted healthcare professionals (nonphysician) (57%), family doctors (70.1%), or mobile phone applications (57.9%), but 53.9% regularly consulted an obstetrician/gynecologist. Table 5 presents the responses to the question: ‘From which source would you best like to get information on the topics of conception and fertility?’. Only one response was allowed. The obstetrician/ gynecologist was their predominant response (76%). Figure 2 illustrates the responses concerning the frequency with which the participants discuss certain topics with a healthcare professional. Almost half of them have never discussed fertility problems/infertility (53.4%), sexually transmitted diseases and fertility (47.4%), or the effect of smoking, alcohol, and drug use on fertility (48.6%) with a healthcare professional. In comparison, 72% have never discussed ART methods.

**Table 4:** Frequency of certain sources used to obtain information on conception and fertility

*Sources*	*Never* *n (%)*	*Some times* *n (%)*	*Often* *n (%)*
Health care professional (non-physician)	466 (57)	250 (30.6)	101 (12.4)
Family doctor	573 (70.1)	178 (21.8)	66 (8.1)
Obstetrician/gynecologist	98 (12.0)	279 (34.1)	440 (53.9)
School/education	382 (46.8)	354 (43.3)	81 (9.9)
Medical websites	113 (13.8)	415 (50.8)	289 (35.4)
Parent/family member	311 (38.1)	360 (44.1)	146 (17.9)
Partner	347 (42.5)	342 (41.9)	128 (15.7)
Friend	168 (20.6)	436 (53.4)	213 (26.1)
Book/magazine	269 (32.9)	393 (48.1)	155 (19.0)
Social media	330 (40.4)	322 (39.4)	165 (20.2)
Mobile phone application	473 (57.9)	244 (29.9)	100 (12.2)

**Table 5:** Preferable source to obtain information on conception and fertility

*Source*	*n (%)*
Health care professional (non-physician)	42 (5.1)
Family doctor	8 (1.0)
Obstetrician/gynecologist	621 (76.0)
School/education	73 (8.9)
Medical websites	38 (4.7)
Parent/family member	6 (0.7)
Partner	3 (0.4)
Friend	1 (0.1)
Book/magazine	11 (1.3)
Social media	6 (0.7)
Mobile phone application	8 (1.0)

## DISCUSSION

In the present study, the knowledge of Greek women of reproductive age, covering reproductive health and conception, tends to be moderate to high. More specifically, the participants had excellent knowledge of the factors that negatively affect fertility and how a woman’s aging affects conception and pregnancy. The last finding agrees with the results of other studies^4^.

Interestingly, more than 95% of women in our sample identified stress as a factor that negatively affects fertility, following other studies^13-16^. On the other hand, data identify significant knowledge gaps and misconceptions in specific fertility awareness topics. Many participants had a limited understanding of the effect of man’s aging on conception and pregnancy, and of the fetal benefits of preconception folic acid administration, in contrast to the results of other studies^16,17^.

Moreover, many women had poor knowledge of practices that may optimize spontaneous conception, such as the correlation of the timing of sexual intercourse with ovulation and conception. Other studies have also identified these significant gaps^16^.

The questions with the smaller proportions of correct responses concerned the increased risk of a miscarriage as the age of the man increases, the negative effect of intense exercise on fertility, and the percentage (75%) of couples who achieve pregnancy within one year of trying. Conversely, the questions with the higher proportions of wrong responses concerned the increased risk of a miscarriage as the age of the man increases and the timing of the sexual intercourse (before ovulation) for conception. The questions and topics with markedly worse results identified in the present study may reflect the areas where attention needs to be given.

Further analysis of the present study’s data revealed that women of higher socioeconomic backgrounds (education, income, and occupation) presented higher knowledge about fertility, aging, ovulation, folic acid, and conception. These results are in contrast with the results of Sarría-Santamera et al.^14^, where none of the socio-demographic factors was correlated with the level of knowledge. Beyond the socioeconomic factors mentioned, age also correlated positively with greater knowledge, as in other studies in the field^6,13,16^. In all cases mentioned above, the score differences between subgroups in the present study were small but statistically significant.

### Childbearing intention and factors that affect the childbearing intention

In accordance with similar studies^10,16^, in the present study, 9 out of 10 women declared an intention to have children, short-term or long-term. This intention was strongly and moderately correlated with demographic and socioeconomic characteristics.

Most participants have set important prerequisites for their decision to have healthy children: a stable partner to share parenthood with, a stable job, and financial security. Several researchers have found the same results^18-26^. On the contrary, and surprisingly, in the present study, the factor ‘completed education’ affects ‘very much’ only half the women sampled. It is difficult to compare the results of the present study with those of similar studies globally due to the heterogeneity and diversity of the samples, different ways to assess fertility awareness, different analytic strategies, and differential findings^8^. Nevertheless, the scale used for the percentage of correct responses (fertility awareness was considered low when <40%, moderate when 40–59%, and high when 60%) has been applied in other studies^8-10,12,13^.

### Beliefs and attitudes toward ART

Almost 6 out of 10 participants said that in case of failure to achieve pregnancy, they would turn to ART, a finding also present in other studies as well^27^. Although this study did not explicitly explore women’s expectations of ART, the international literature on this topic is consistent with the low results in ART knowledge (Q32 and Q33, Figure 1). Moreover, we identified a significant percentage of women who were uninformed about the accessibility, the cost, and the treatments offered in ART, similar to other studies^28-30^. Even in public health units, ART is not fully covered by public health insurance in Greece. More specifically, health insurance covers medication expenses and some medical tests, while an allowance is given to the couple in order to cover part of the expenses. Generally, Greece has one of the lowest costs of IVF treatment compared with other European countries. It offers a clear legislative framework governing IVF that allows access to ART for women (aged <54 years) and men (regardless of age).

Furthermore, in Greece, a national authority governs the scientific, legal, and moral frame in which all clinics and organizations related to ART are functioning. The Greek National Authority of Assisted Reproduction can also play a significant role in clearing up the public’s misconceptions regarding ART by organizing various activities and actions in the future.

### Sources to obtain information on conception and fertility

From the wide range of sources to obtain information on conception and fertility, the present study’s participants seem to prefer consulting obstetricians/gynecologists and medical websites. These findings are consistent with other studies^29,31,32^. Nearly 50% declared they have never obtained information from school/education. This finding can be attributed to the absence of fertility education in the curriculum at any level of Greek education. Likewise, social media and mobile phone applications seem to contribute less than expected, and our findings here contrast with other studies’ results^6,16,33,34^. On the other hand, as was expected, most respondents would prefer the obstetrician/ gynecologist as the top source for information on conception and fertility (76%), consistent with the results of other studies^16^.

The interesting and surprising finding that merits discussion is that almost half of the participants had never discussed topics related to conception, fertility, infertility, ART, and prenatal screening with a healthcare professional. This finding seems to contradict the percentage of the participants (90%) who intended to have children in the future or were trying to conceive during the study.

### Strengths and limitations

The strengths of the present study include a diverse population of reproductive-age women aged 18–40 years. Moreover, the questionnaire had many questions on reproductive and fertility knowledge and thus provided a detailed perception of the participants’ background knowledge of the subject. Limitations of the study include the moderate sample size and the potential for participants to have unique characteristics compared with the general population, owing to the online survey methodology and the non-random, convenience sampling method. However, the sample was heterogeneous regarding age, education level, and economic status. Moreover, because 9 out of 10 participants declared an intention to have children, it can be assumed that they may be more interested and have invested in future fertility, which is why they have better knowledge than other populations^8^. Furthermore, a validated instrument (standardized questionnaire) was not used for the knowledge assessment, a general limitation in research focusing on fertility awareness^22,23^. This finding makes it difficult to compare the present research with other research in the field.

### Addressing the aspects of sexual and fertility education in Greece

In Greece, it has been more than three decades since the topic of sexual and fertility education was raised. Still, it has not been implemented in practice since it has not been included as a compulsory subject in the curriculum at any education level. Optionally, primary healthcare workers can be invited by schools to give a 1-hour lecture about reproductive health. The available material from the Hellenic Ministry of Health for this purpose is limited and offers basic knowledge on anatomy, the menstrual cycle, contraception, and sexually transmitted diseases. The phenomenon of schools’ sexual education curricula being generally focused on preventing pregnancy is global^34^. Moreover, there is no strategic planning on fertility education, as has been implemented in other countries^7,35,36^. The Ministry of Health and National scientific societies (medical, midwifery, and nursing) organize, from time to time, various activities and actions, for example, informing the public during the ‘fertility day’ or hosting a conference for healthcare professionals. However, the effectiveness of these initiatives has not been assessed in depth.

Future research should assess the primary sources of women’s and men’s fertility knowledge and determine if and how increased knowledge translates into action. It is also important to determine whether increased knowledge of fertility and family planning options reduces the growing trend towards delayed childbearing and later parenting, resulting in more informed and satisfying reproductive decision-making.

## CONCLUSIONS

We assessed the knowledge of Greek women aged 18–40 years regarding reproductive health and conception. Significant knowledge gaps and misconceptions were identified in specific fertility topics. Almost half of the participants in the present study had never discussed topics related to conception, fertility, infertility, ART, and prenatal screening with a healthcare professional. This result underlines the need for strategic involvement of healthcare professionals in fertility education and awareness. Targeted educational and primary care intervention development may improve fertility awareness and informed decisions about childbearing.

## Supplementary Material



## Data Availability

The data supporting this research are available from the authors on reasonable request.
