# Supplementary material for: Conception and fertility: A survey examining the practices, attitudes, and knowledge of reproductive-age Greek women
Source: Eur J Midwifery. 2025 Mar 26;9:10.18332/ejm/200843. doi: 10.18332/ejm/200843 (PMC12527117; doi:10.18332/ejm/200843)
Supplement: Supplementary file 1 [file EJM-9-17-s1.pdf]

Table Sup1. Score per subgroup according to age, marital status, education, occupation, personal monthly income, residence and childbearing intention

		Mean	SD	P value
**Age**	18-25 years	18.2	4.7	<0.001
	26-30 years	19.6	4.6	
	31-35 years	20.1	4.6	
	36-40 years	21.3	4.8	
**Marital status**	Married	21.0	4.7	<0.001
	In a stable relationship	19.4	4.6	
	In a non-stable relationship	19.0	5.1	
	Single	18.6	4.8	
**Education**	High School	17.5	4.2	<0.001
	Technical education	18.8	4.8	
	College / University	19.6	4.6	
	MSc / PhD	20.6	4.9	
**Occupation**	Employee, Public sector	21.3	4.4	<0.001
	Employee, Private sector	20.2	4.6	
	Self-employed	19.0	4.6	
	Student	17.9	4.6	
	Unemployed	17.9	5.3	
**Personal monthly income**	0 €	17.3	4.8	<0.001
	1-500 €	18.6	4.4	
	501-1000 €	20.0	4.8	
	1001-2000 €	21.3	4.4	
	>2001 €	21.0	4.2	

**Residence**	Athens / Thessaloniki	19.9	4.8	0.139
	Urban area	18.8	5	
	Semi-urban area	19.1	4.5	
	Rural area	18.9	4.7	
	Abroad	19.6	4.9	
**Childbearing intention**	I don't intend to have children	20.0	4.6	<0.001
	I intend to have children in the future	19.3	4.8	
	Trying to conceive	21.6	4.5	

Values are presented as numbers ± standard deviation (SD).

© 2025 Saranti E. et al.
